# Deep intronic deletion in intron 3 of *PLP1* is associated with a severe phenotype of Pelizaeus-Merzbacher disease

**DOI:** 10.1038/s41439-021-00144-y

**Published:** 2021-04-01

**Authors:** Keiko Yamamoto-Shimojima, Hiroyuki Akagawa, Kumiko Yanagi, Tadashi Kaname, Nobuhiko Okamoto, Toshiyuki Yamamoto

**Affiliations:** 1 Department of Transfusion Medicine and Cell Processing, Tokyo Women’s Medical University, Tokyo, 162-8666 Japan; 2 Institute of Medical Genetics, Tokyo Women’s Medical University, Tokyo, 162-8666 Japan; 3 Tokyo Women’s Medical University Institute of Integrated Medical Sciences, Tokyo, 162-8666 Japan; 4 Department of Genome Medicine, National Center for Child Health and Development, Tokyo, 157-8535 Japan; 5 Department of Medical Genetics, Osaka Women’s and Children’s Hospital, Osaka, Japan

**Keywords:** Gene amplification, Genetics research

## Abstract

Recently, altered *PLP1* splicing was confirmed as a genetic cause of hypomyelination of early myelinating structures (HEMS). A novel deep intronic deletion in intron 3 of *PLP1* (NM_000533.5: c.453+59_+259del) was identified, and an in vitro minigene assay detected abnormal splicing patterns. However, the clinical and radiological findings of the patient were compatible with a severe phenotype of Pelizaeus-Merzbacher disease rather than HEMS, which may be due to undetected abnormal *PLP1* splicing.

Pelizaeus-Merzbacher disease (PMD: MIM #312080) is one of the major types of hypomyelinating leukodystrophy^1^. The gene responsible for this disease is the proteolipid protein 1 gene (*PLP1*), which is located on Xq22^2^. Thus, PMD is recognized as an X-linked recessive disorder. Approximately two-thirds of PMD patients show microduplications, including in *PLP1*, and the other patients show nucleotide alterations in *PLP1*^3^. Previously, many variants have been reported as pathogenic. Although missense variants are the major type of variants, a number of splicing variants have also been reported^4–7^. In particular, variants in intron 3 (IVS3) have been detected to cause unique radiological findings recognized as hypomyelination of early myelinating structures (HEMS)^8,9^. Here, we report a novel intronic variant identified in a patient with typical PMD features rather than HEMS.

At present, the male patient is 4 years and 6 months old. The boy was born at full term with a birth weight of 2886 g, length of 51.0 cm, and occipitofrontal circumference (OFC) of 32.0 cm. Although there was no remarkable episode during early infancy, pendular nystagmus was noted at 3 months of age. He has never achieved head control and is still bed ridden. There is no verbal communication. There is no stridor, and he can swallow paste food. Owing to the acceleration of the deep tendon reflex, spastic paraplegia was diagnosed. Brain magnetic resonance imaging (MRI) performed at 2 years and 9 months showed diffuse high intensity of the white matter in T2-weighted images in association with the maturation of myelination in the corpus callosum, indicating the typical hypomyelination pattern observed in patients with PMD (Fig. 1A and Supplemental Fig. S1). There were no other abnormalities, including no dilatation of the lateral ventricles. From these findings, PMD was suspected. At present, his height is 93 cm (−2.7 SD), his weight is 11.4 kg (−2.5 SD), and his OFC is 48.5 cm (−1.2 SD), indicating growth deficiency but no microcephaly. Conventional FISH analysis targeting *PLP1* did not demonstrate *PLP1* duplication. Thus, a comprehensive analysis was planned.

**Fig. 1:**
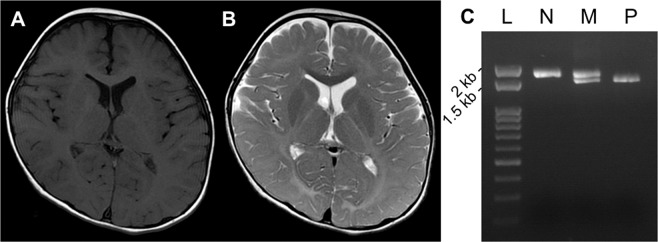
Information of the present patient. T1- (**A**) and T2- (**B**) weighted brain magnetic resonance imaging examined at 2 years of age shows T2-high intensity in the deep white matter. A normal myelination pattern is shown in the corpus callosum. There is no structural abnormality. **C** Genomic PCR for exons 3–4 of *PLP1*. In comparison with the normal control (N), an abnormal short band is shown in the patient (P). The mother (M) exhibits both bands, indicating that she is a heterozygous carrier of this variant. L; Gene Ladder 100 (Nippon Gene, Tokyo, Japan).

This study was performed in accordance with the Declaration of Helsinki. We obtained permission from the ethics committee of the institution. The family of this patient provided written informed consent after genetic counseling. Blood samples were obtained from the patient and his parents. Subsequently, genomic DNA was extracted using the standard method, and whole-exome sequencing using trio samples, including parental samples, was performed as described previously^10^. However, no possible candidate variant related to leukodystrophy was identified. The diagnostic strategy was then reconsidered. Chromosomal microarray testing was performed, and no pathogenic copy number aberration was identified. Further, all coding exons of *PLP1* were planned to be reanalyzed by standard PCR-Sanger sequencing. In this process, we could not obtain a PCR amplicon of exon 3, although the other six exons were successfully amplified. Next, we used primers for neighboring exons, including exon 3 forward and exon 4 reverse primers. As a result, a shorter PCR band was confirmed by agarose gel electrophoresis (Fig. 1B). His mother showed both normal and short bands, suggesting that she was a carrier of this *PLP1* variant. Sanger sequencing using a PCR amplicon of the short band confirmed a 201-bp deletion in IVS3 59 bp from the exon/intron boundary (NM_000533.5: c.453+59_+259del). Retrospective analysis of the data obtained through whole-exome sequencing showed that there was no mapped read in the identified deletion region, indicating the nullisomy of this region (Supplemental Fig. S2).

To confirm whether this deletion may cause splicing abnormalities, RNA expression from EB virus-transformed leukocytes was analyzed by nested RT-PCR according to a previously reported method^11^. However, we could not obtain a PCR amplicon due to the insufficient expression level of *PLP1* in the cells. Thus, an in vitro assay was tried as an alternative method. For DNA cloning of the genomic region from IVS3 (−110) to IVS4 (+88) of *PLP1*, a set of primers (PLP1_insF; TAACTCGAGTAGCCTTGTTAAGGTGCTCGCT, Xba1_PLP1_insR; ATATCTAGACACCACCCTCCTTACACTAAGA) including the restricted enzyme sites Xho1 and Xba1 (indicated by underbars) was designed. Samples from a normal control with the wild-type allele and the genomic DNA of the present patient were used as templates. Then, two PCR amplicons of 1700 bp and 1499 bp were obtained. These amplicons underwent Xho1 and Xba1 restriction digestion and were cloned into the pET 01 Exontrap vector (MoBiTec GmbH, Gottingen, Germany) (Fig. 2A).

**Fig. 2:**
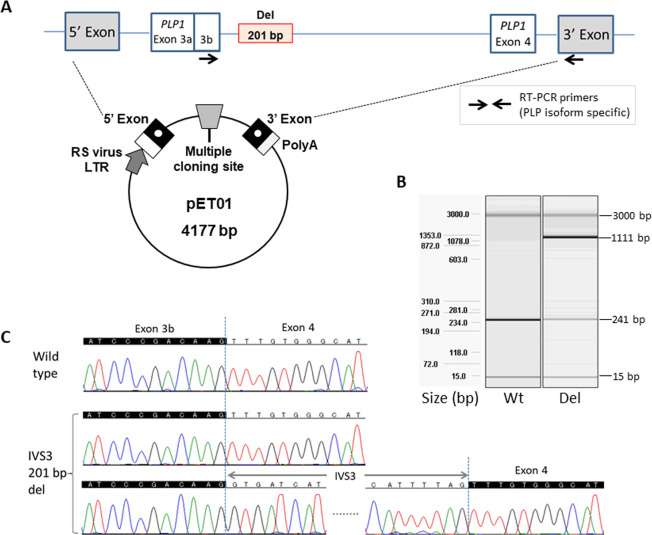
Experimental procedures of the minigene assay in this study. **A** Physical map of the vector used in this study. A partial sequence of *PLP1* was cloned into multiple cloning sites. **B** In comparison with the control, QIAxcel semiquantitative RT-PCR shows 67.0% reduced expression of *PLP1* in addition to aberrant large products. **C** Sanger sequencing confirms intron 3 (IVS3) retention in mRNA.

The successfully constructed vectors were transfected into COS7 cells by lipofection. After 24 h of incubation, total RNA was extracted, and RNA expression from mini-*PLP1* was examined by RT-PCR. Two different isoforms, *PLP1* and *DM20*, are expressed from *PLP1*. *DM20* mRNA does not include exon 3b, which is included in *PLP1* mRNA. Through the use of the primers designed in the vector region, only *DM20* was detected, and *PLP1* expression could not be detected as previously reported^12^. Thus, we focused only on *PLP1*. For the analysis of *PLP1* splicing, a 5ʹ RT-PCR primer (PLP_isoF; GAGCGGGTGTGTCATTGTTT) was designed in an exon 3b-specific region. On the other hand, a 3ʹ RT-PCR primer (PLP_isoR; CTCCACCCAGCTCCAGTTGT) was used in the 3ʹ exon region in the vector. RT-PCR amplicons were analyzed by capillary electrophoresis using a QIAxcel Advanced System (Qiagen, Venlo, The Netherlands). The normal splicing pattern was confirmed in the wild type. On the other hand, the deletion mutant showed a large RT-PCR product in addition to the wild-type product and multiple faint bands (Fig. 2B). The expression level of wild-type RNA was reduced by 67.0%. Sanger sequencing of the large RT-PCR product confirmed that the full sequence of IVS3 was included between exon 3b and exon 4, suggesting that this intron was not normally spliced out (Fig. 2C). Because wild-type IVS3 is 1071 bp in length, the retained intron in this patient is considered to be 870 bp in length (201 bp shorter than wild type) and would insert nine amino acids and a premature termination codon [p.(Lys151_Phe152insValIleIleLeuArgLieLeuTrpGlinTer)]. Thus, the expression of abnormal mRNA related to the large RT-PCR product, which includes this retained intron, would be prevented by nonsense-mediated decay.

Previously, many splicing mutations have been reported^4–7^. In the splicing donor site of IVS3, three variants were reported in 2000^13^. Two of them affected the consensus donor site (IVS3+2T>C and IVS3+4A>G). The remaining deletion was a 19-bp deletion in the enhancer region (IVS3+28_+46del), which reduced the expression of *PLP1* but not *DM20* (ref. ^14^). This unbalanced expression of *PLP1* and *DM20* caused a milder phenotype of PMD. In 2015, mutations in exon 3B and IVS3 were identified in patients with HEMS^9^, which is characterized by unique hypomyelination patterns specifically pronounced in early myelinating structures, including alternating T2 hyperintense–hypointense–hyperintense stripes in the posterior limb of the internal capsule^8^. The identified mutations in HEMS are predicated to cause abnormal splicing in *PLP1* but not in *DM20*. Thus, unbalanced expression of *PLP1* and *DM20* is also considered the pathogenesis of HEMS. Because all patients with HEMS are able to achieve unsupported walking^8^, the clinical features are milder than those with typical PMD.

In this study, a 201-bp deletion (IVS3+59_+259del) was identified in the region neighboring the abovementioned 19-bp deletion (IVS3+28_+46del). Furthermore, this 201-bp deletion removed a 5ʹ-long-distance interaction (LDI) structure^15^, in which two noncoding mutations have been identified in patients with HEMS^9^. In this study, an in vitro mini-*PLP1* gene assay detected abnormal splicing patterns similar to HEMS. However, the clinical features of the present patient were more severe than those of the patients with HEMS, and the radiological findings were also different from those of patients with HEMS. On the basis of these points, we believe that the previously undetected underlying abnormal *PLP1* splicing is the cause of the severe clinical features in this patient. The limitation of this study is that the mini-*PLP1* gene cloned into the vector contained only exons 3 and 4. Thus, some other splicing abnormalities may have been missed.

In this study, we failed to detect a deep intronic deletion by initial whole-exome sequencing. More attention should be paid to the relevance of deep intronic variants in genetic diseases^16,17^.

## Supplementary information

Supplemental Figure S1

Supplemental Figure S2

## Data Availability

The relevant data from this Data Report are hosted at the Human Genome Variation Database at 10.6084/m9.figshare.hgv.2984.
