# Supplementary material for: Deep intronic deletion in intron 3 of PLP1 is associated with a severe phenotype of Pelizaeus-Merzbacher disease
Source: Hum Genome Var. 2021 Apr 1;8:14. doi: 10.1038/s41439-021-00144-y (PMC8016919; doi:10.1038/s41439-021-00144-y)
Supplement: Supplementary file 1 — Supplemental Figure S1 [file 41439_2021_144_MOESM1_ESM.pdf]

Supplemental Figure S1. Additional brain MRI findings of the patient

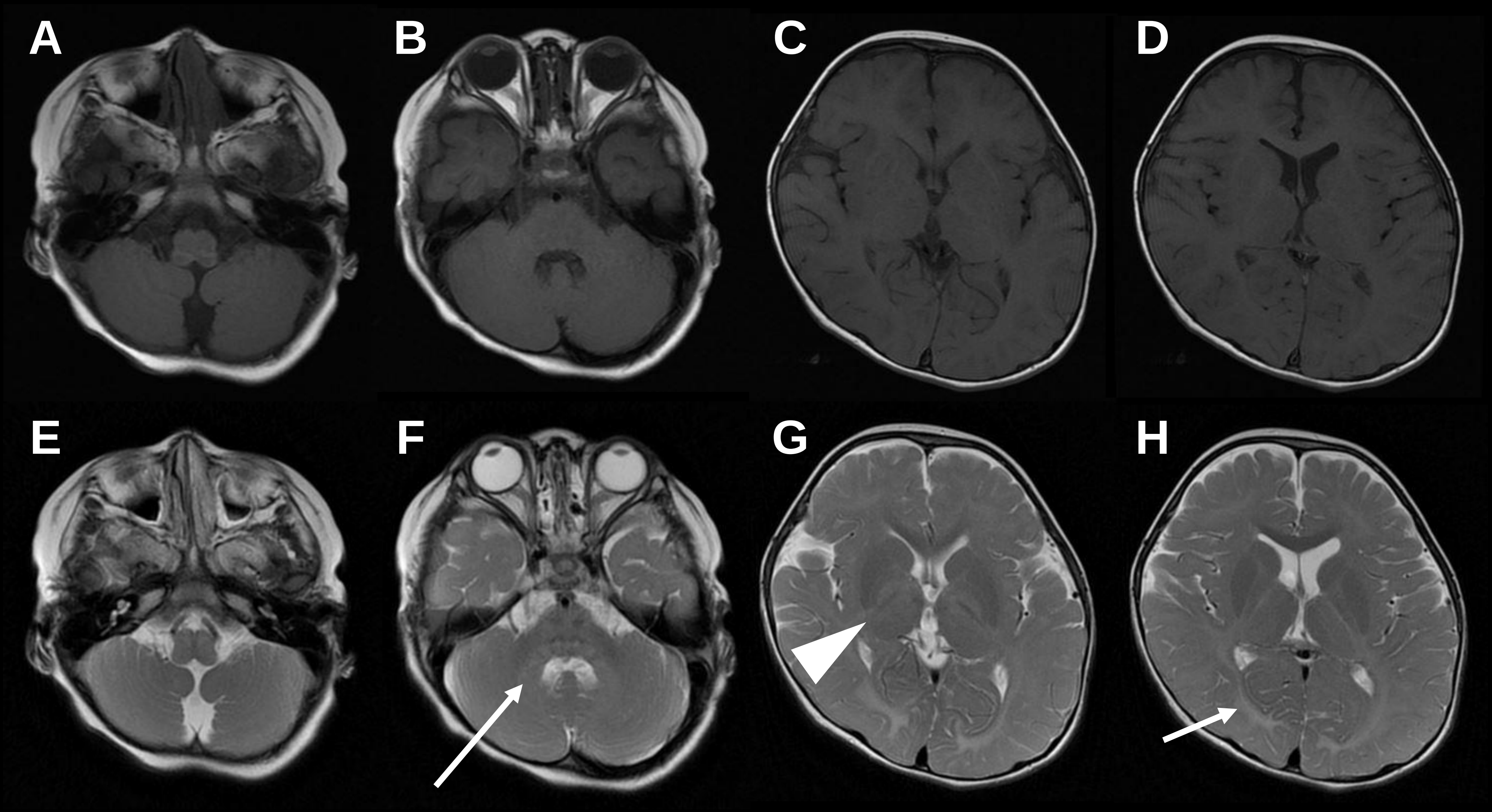

Axial images of T1- (A-D) and T2- (E-H) weighted MRI. T2-hypointensity regions are indicated by arrows. The alternating T2 hyperintense-hypointense-hyperintense stripes in the posterior limb of the internal capsule (typical finding in HEMS) is not observed (an arrow head).

Supplemental Figure S1. Additional brain MRI findings of the patient (continue)

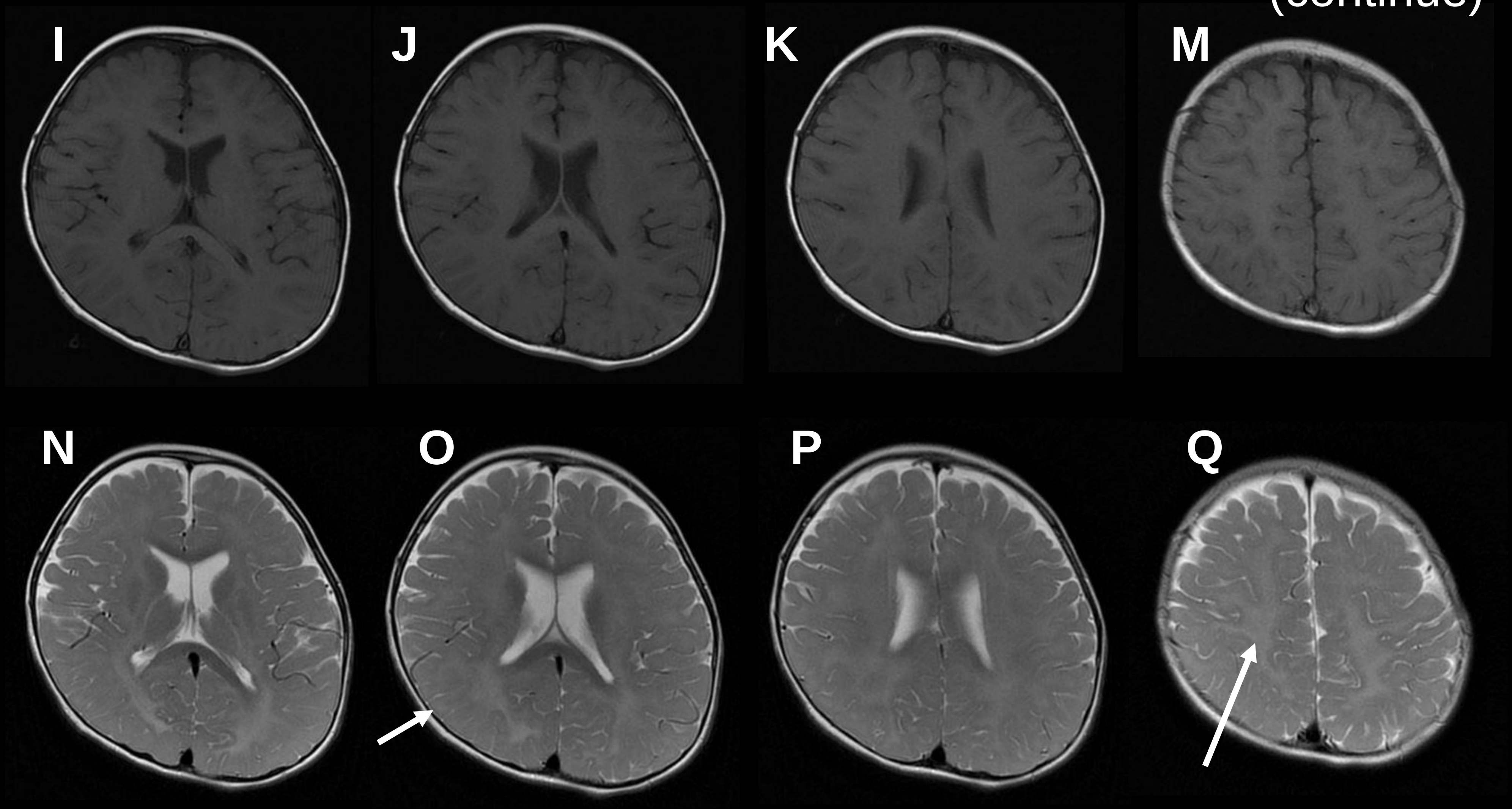

Axial images of T1- (I-M) and T2-(N-Q) weighted MRI. T2 hyperintensity is observed in the subcortical white matter (O: arrow) and the periventricular white matter region (Q: arrow).
