# Supplementary material for: Deep intronic deletion in intron 3 of PLP1 is associated with a severe phenotype of Pelizaeus-Merzbacher disease
Source: Hum Genome Var. 2021 Apr 1;8:14. doi: 10.1038/s41439-021-00144-y (PMC8016919; doi:10.1038/s41439-021-00144-y)
Supplement: Supplementary file 2 — Supplemental Figure S2 [file 41439_2021_144_MOESM2_ESM.pdf]

Supplemental Figure S2.
Demonstration of the results of whole exome sequence

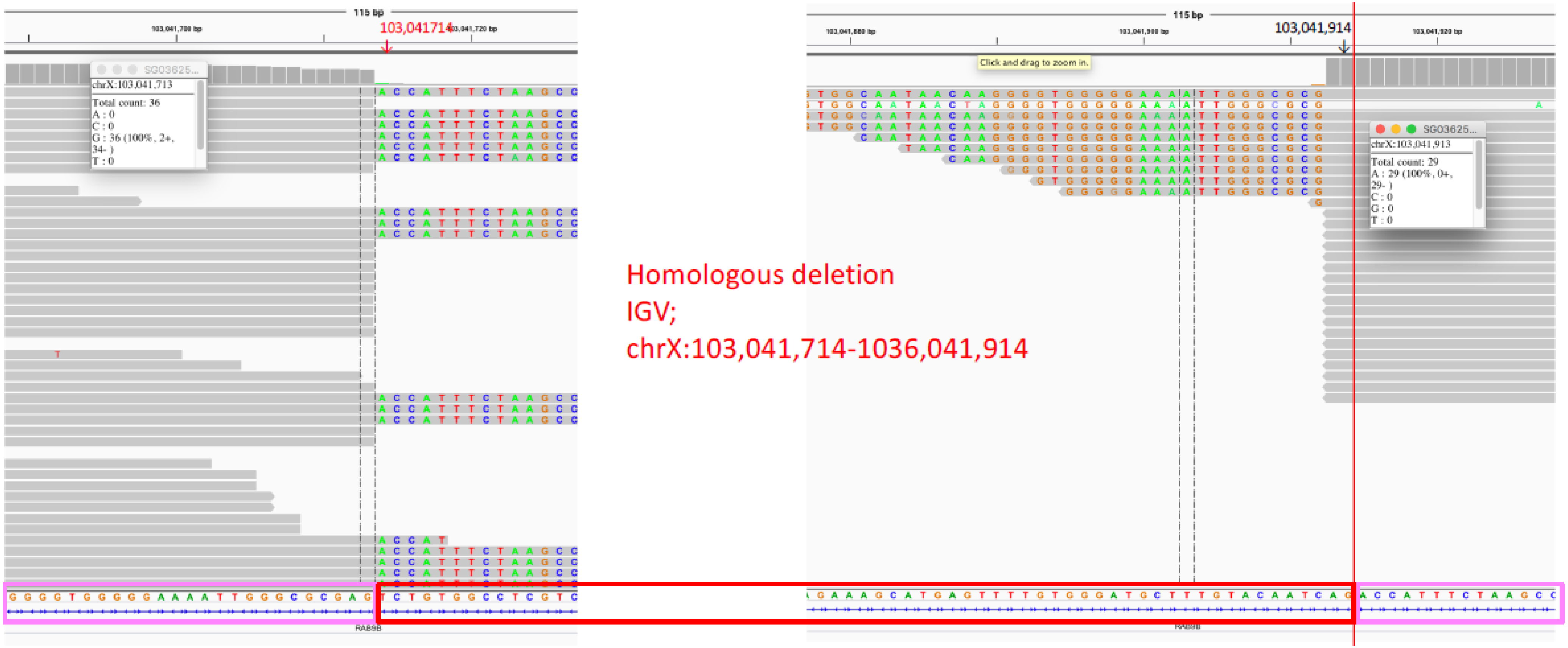

The identified deletion region is retrospectively analyzed by Integrative Genomics Viewer (IGV; https://software.broadinstitute.org/software/igv).
